# Acute mesenteric haematoma and hematoperitoneum following a coughing episode induced by COVID-19

**DOI:** 10.1093/jscr/rjad450

**Published:** 2023-08-08

**Authors:** Charles Carey, Maryam Khatoon, Dare Seriki, Anselm Agwunobi

**Affiliations:** The General Surgery Department, Wythenshawe Hospital, Manchester University NHS Foundation Trust Cobbett House Manchester Royal Infirmary, Manchester M13 9PL, UK; The General Surgery Department, Wythenshawe Hospital, Manchester University NHS Foundation Trust Cobbett House Manchester Royal Infirmary, Manchester M13 9PL, UK; The General Surgery Department, Wythenshawe Hospital, Manchester University NHS Foundation Trust Cobbett House Manchester Royal Infirmary, Manchester M13 9PL, UK; The General Surgery Department, Wythenshawe Hospital, Manchester University NHS Foundation Trust Cobbett House Manchester Royal Infirmary, Manchester M13 9PL, UK

**Keywords:** mesenteric haematoma, haematoperitoneum, COVID-19

## Abstract

Acute mesenteric haematoma (AMH) is a rare condition and established causes include blunt trauma, aneurysmal rupture, acute pancreatitis and anticoagulant use. A male patient in his 50s presented with abdominal pain and loss of consciousness that was immediately preceded by a prolonged coughing episode. A computed tomography (CT) abdomen-pelvis revealed two acute mesenteric haematomas and haematoperitoneum and admission swabs diagnosed coronavirus disease 2019 (COVID-19). The patient had no other acute clinical issues and was not taking anticoagulants. The haematomas were managed conservatively and a follow up computed tomography (CT) 4 weeks post-discharge revealed significant improvement. No clear vessel was identified as the source of the bleed in any of the investigations. This case represents a rare instance of AMH and haematoperitoneum with no established cause. We theorize that the combination of the patient’s systemic response to COVID-19 and raised intra-abdominal pressure caused by coughing contributed to the bleeding.

## INTRODUCTION

Acute mesenteric haematoma (AMH) is a rare condition and established causes include trauma, aneurysm rupture, acute pancreatitis and treatment with anticoagulants [1, 2]. In uncomplicated cases, conservative management has been employed successfully [2, 3]. Coronavirus disease 2019 (COVID-19) is known to cause multiple organ pathologies including venous thromboembolism, vasculitis and cardiac myositis [4]. Although thromboembolic events are more commonly associated with COVID-19, large studies have shown that major haemorrhage is a rare but significant risk during COVID-19 infection [5, 6]. Other case reports have also highlighted instances of major haemorrhage associated with COVID-19 [7–9].

This report describes a patient who suffered two episodes of AMH and haematoperitoneum following a prolonged coughing episode caused by COVID-19. Since the patient lacked any of the established causes of AMH, it is possible that his bleed occurred secondary to the systemic effects of COVID-19.

## CASE REPORT

A male patient in his 50s was admitted with severe abdominal pain and a brief loss of consciousness that was immediately preceded by a prolonged coughing episode. His initial heart rate was 113 bpm and blood pressure was 115/81 mmHg. His lying and standing blood pressure measurements revealed a postural drop from 103/74 to 67/40 mmHg and he was admitted to resus. On examination the patient displayed left upper quadrant pain and a FAST ultrasound scan revealed free fluid in the abdomen. There was no per rectal bleeding and the patient’s blood tests revealed haemoglobin (Hb) = 126 g/L, white cells = 16.4 x 10^9^ cells/L, CRP = 5 mg/L, INR = 1.0, lactate = 1.5 mmol/L and pH = 7.37. The patient’s past medical history included a previous right-sided inguinal hernia and depression. His medications included 30 mg mirtazapine taken once daily and he had no known drug allergies.

**Figure 1:**
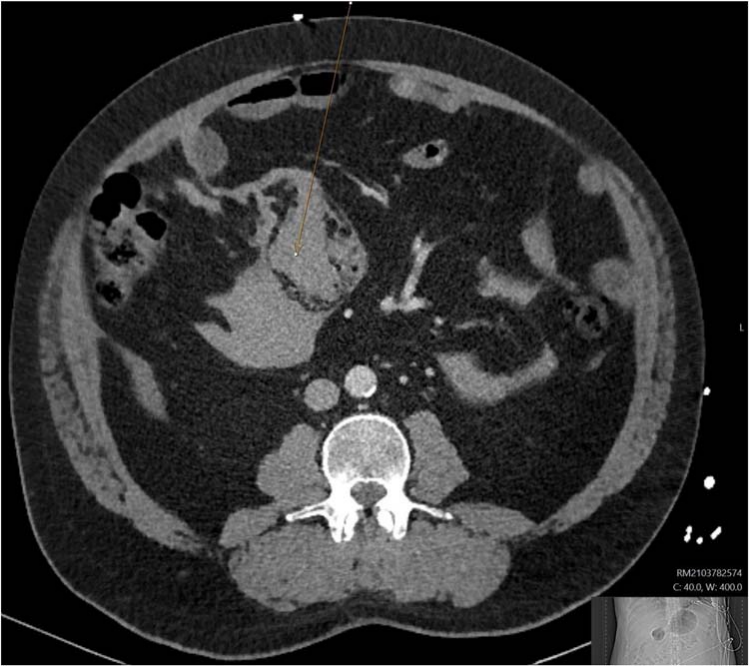
CT angiogram showing a large acute mesenteric haematoma. Figure 1 is taken from an axial section of the patient’s initial CT angiogram of the abdomen and pelvis. The arrow shown highlights an area of high-density fluid representing a 104 x 62 mm acute mesenteric haematoma.

**Figure 2:**
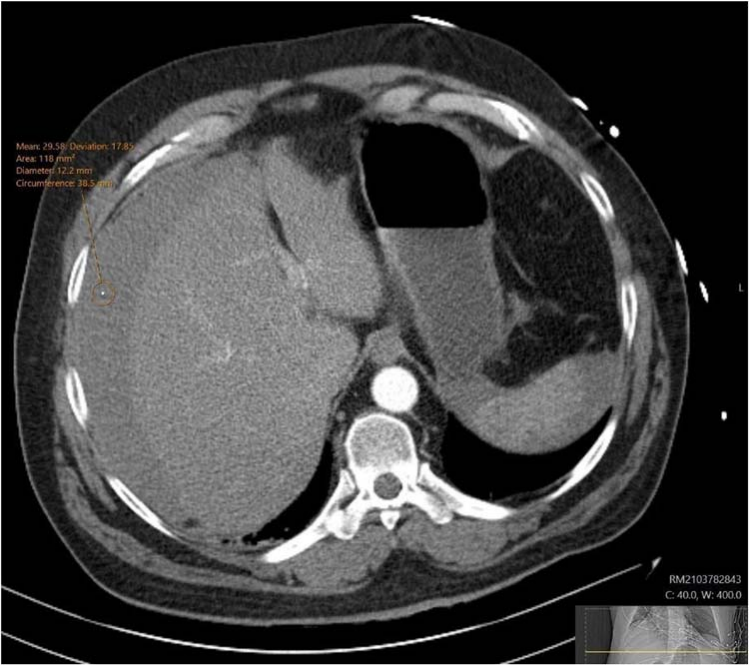
CT angiogram showing a peri-hepatic haematoma. Figure 2 is an axial section taken from the patient’s initial CT angiogram of the abdomen and pelvis. A large crescent shaped peri-hepatic haematoma is highlighted by the arrow displayed.

**Figure 3:**
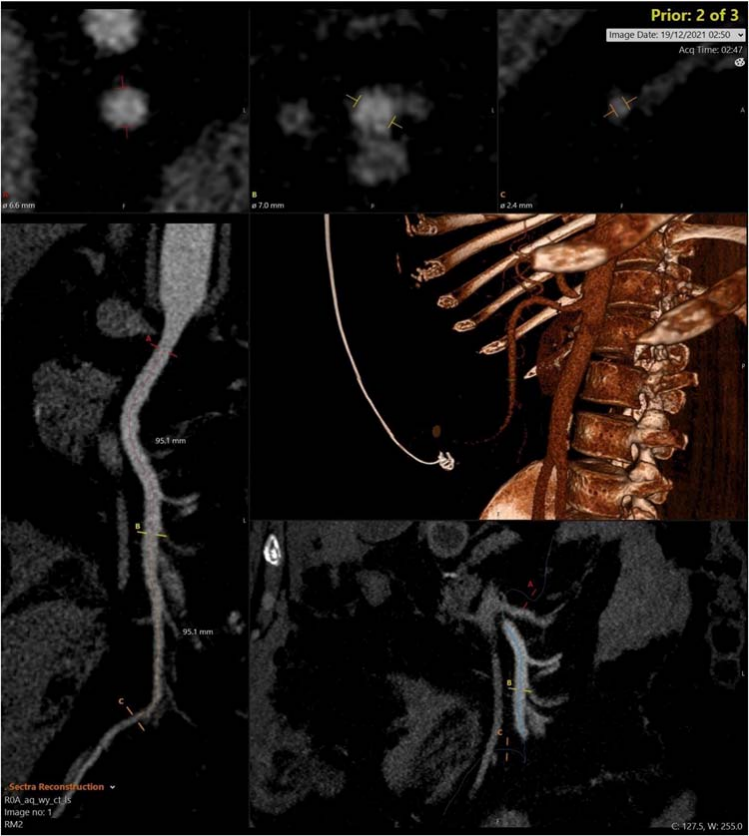
Curved reprogramming of the initial CT angiogram. Curved reprogramming of the patient’s initial CT angiogram showed no evidence of an aneurysm within the superior mesenteric artery, effectively ruling this out as a cause of the patient’s acute bleed.

A CT abdomen-pelvis (CT-AP) revealed an acute haematoma inferolateral to the third part of the duodenum and another at the root of the mesentery. High density fluid related to the mesenteric bleed was also seen extending from the right para-colic gutter into the pelvis and in the peri-hepatic region. The patient was admitted under general surgery, stabilized with intra-venous fluid resuscitation, co-amoxiclav and metronidazole and cross matched for four units of blood. A CT-angiogram demonstrated acute haemorrhage throughout the abdomen with a 104 x 62 mm haematoma arising centrally at the level of L2 within the mesenteric fat (Fig. 1). The haematoma did not appear to originate from any specific mesenteric vessels but was related to some mid and distal jejunal loops. The angiogram also showed discontinuity when tracing the proximal and mid jejunal loops inferiorly, extensive haemorrhagic peritoneal fluid around the liver extending inferiorly (Fig. 2) and a small amount of peri-splenic haemorrhagic fluid. Curved reformatting of the angiogram revealed no abnormalities within the superior mesenteric artery (Fig. 3).

On Day 2 of admission the patient’s blood tests revealed a significant Hb decrease to 83 g/L, which improved to 92 g/L following transfusion with 1 unit of packed red cells. Since he remained clinically stable after resuscitation, no procedures were considered necessary for definitive management. He was diagnosed with COVID-19 following positive PCR testing and suffered no significant respiratory complications throughout admission. A repeat single-phase CT-AP showed pooled haemorrhages in the pelvis and paracolic gutters not seen previously and an interval increase in the peri-splenic haematoma. The scan also revealed relatively thick proximal loops of jejunum, suggesting that the bleeding source came from a vessel supplying this area. No single vessel, however, could be identified as the source of the bleed.

**Figure 4:**
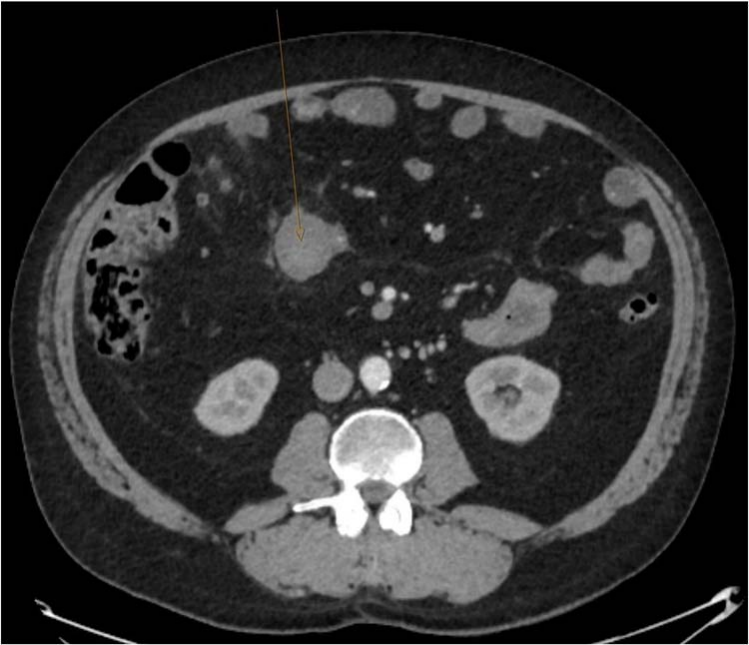
Follow up CT angiogram showing improvement in mesenteric haematoma. Figure 4 is an axial section from the patient’s CT scan performed 4 weeks following discharge. The arrow highlights the patient’s liquefying mesenteric haematoma, which decreased from 104 x 62 mm to 60 x 40 mm. Combined with the improvement in the other areas of intra-abdominal bleeding and the patient’s overall clinical state, this image represents a significant improvement.

**Figure 5:**
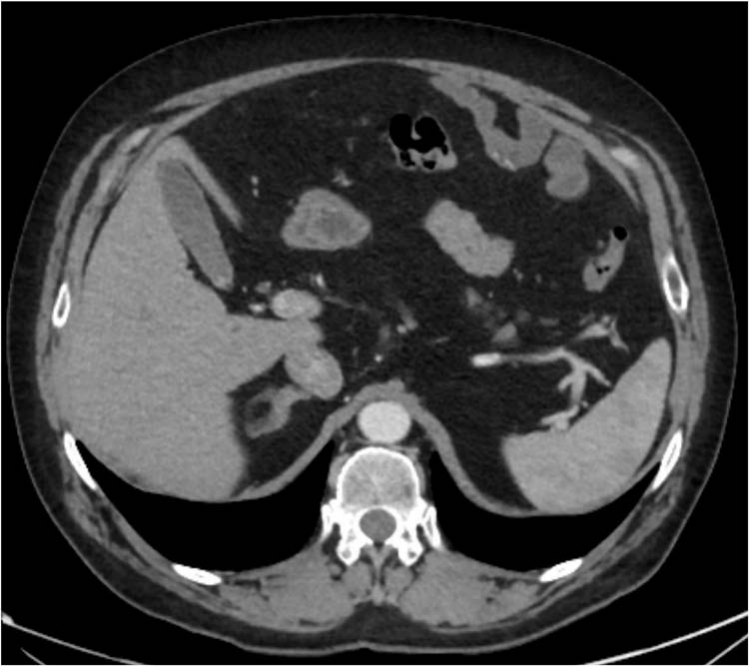
Follow up CT angiogram showing improvement in perihepatic haematoma. Figure 5 is another axial section from the patient’s CT angiogram performed 4 weeks following discharge and shows resolution of the perihepatic haematoma seen previously. Together with the other sections of the CT angiogram and the patient’s overall clinical state, the image represents a significant improvement in the patient’s intra-abdominal bleeding.

During admission the patient’s abdominal pain gradually improved, his Hb increased and he had no further coughing episodes. The patient was therefore stepped down to oral antibiotics and considered safe for discharge. A CT-AP four weeks post-discharge demonstrated a persisting 60 x 40 mm liquefying haematoma in the small bowel (Fig. 4), resolution of the peri-hepatic haematoma (Fig. 5) and interval reduction of the peri-splenic and pelvic haematomas. Given these improvements and the patient’s lack of significant symptoms, a plan to actively survey his progress was made.

## DISCUSSION

It is exceedingly rare for AMH to occur in the absence of blunt trauma to the abdomen, superior mesenteric artery aneurysm or use of anticoagulants [1, 2]. Our patient however, displayed none of these risk factors during his acute and follow up investigations or in his prior medical history.

Large studies by Pavlov *et al*. and Altschul *et al*. found that the incidence of cerebral haemorrhage during COVID-19 infection was 0.25% and 0.6%, respectively [5, 6]. Furthermore, Singh *et al*. reported large bleeds within the chest wall and lower limbs of four patients with COVID-19 and Mohamed *et al*. presented a case in which infection with the virus was associated with major upper GI bleeding [7, 8]. Shah *et al*. also found that 8.0% of COVID-19 patients admitted to intensive therapy unit (ITU) suffered a haemorrhagic event, with the most common site being within the gastrointestinal tract [9]. Together these studies suggest that haemorrhage is a rare but significant risk during COVID-19 infection.

Prolonged coughing is an established symptom of COVID-19 and leads to raised intra-abdominal pressure (IAP) due to the coordinated contraction of thoracic, abdominal and pelvic muscles [10]. Raised IAP secondary to extensive coughing has been associated with spontaneous sheath haematoma and it is possible that the prolonged coughing in our case contributed to his bleeding [11]. COVID-19 is also known to enter cells via the ACE2 receptor, which becomes downregulated following viral invasion [12]. The depletion of this protein may increase the risk of haemorrhage by promoting interaction between angiotensin 2 and AT1 receptors, resulting in localized hypertension, or by leading to endothelial dysfunction [5].

In conclusion, the absence of established causes in this case raises the possibility that the AMH and haematoperitoneum occurred secondary to raised IAP induced by prolonged coughing as well as the systemic responses to COVID-19. This case and other studies highlight the risk of acute bleeding during COVID-19 infection and the need for greater awareness of this complication.

## Data Availability

The data used in this publication is only available to readers in this article and may not be accessed through other means in order to protect patient confidentiality. There are no additional data sets or figures associated with this article that may be provided.
